# GABA and Glutamate in hMT+ Link to Individual Differences in Residual Visual Function After Occipital Stroke

**DOI:** 10.1161/STROKEAHA.123.043269

**Published:** 2023-07-21

**Authors:** Hanna E. Willis, I. Betina Ip, Archie Watt, Jon Campbell, Saad Jbabdi, William T. Clarke, Matthew R. Cavanaugh, Krystel R. Huxlin, Kate E. Watkins, Marco Tamietto, Holly Bridge

**Affiliations:** 1 Wellcome Centre for Integrative Neuroimaging, Nuffield Department of Clinical Neurosciences (H.E.W., I.B.I., A.W., J.C., S.J., W.T.C., H.B.), University of Oxford, United Kingdom.; 2 Wellcome Centre for Integrative Neuroimaging, Department of Experimental Psychology (K.E.W.), University of Oxford, United Kingdom.; 3 Flaum Eye Institute and Center for Visual Science, University of Rochester, NY (M.R.C., K.R.H.).; 4 Department of Psychology, University of Torino, Italy (M.T.).; 5 Department of Medical and Clinical Psychology, and CoRPS—Center of Research on Psychology in Somatic Diseases—Tilburg University, the Netherlands (M.T.).

**Keywords:** hemianopia, magnetic resonance imaging, quality of life, stroke, survivors

## Abstract

**BACKGROUND:**

Damage to the primary visual cortex following an occipital stroke causes loss of conscious vision in the contralateral hemifield. Yet, some patients retain the ability to detect moving visual stimuli within their blind field. The present study asked whether such individual differences in blind field perception following loss of primary visual cortex could be explained by the concentration of neurotransmitters γ-aminobutyric acid (GABA) and glutamate or activity of the visual motion processing, human middle temporal complex (hMT+).

**METHODS:**

We used magnetic resonance imaging in 19 patients with chronic occipital stroke to measure the concentration of neurotransmitters GABA and glutamate (proton magnetic resonance spectroscopy) and functional activity in hMT+ (functional magnetic resonance imaging). We also tested each participant on a 2-interval forced choice detection task using high-contrast, moving Gabor patches. We then measured and assessed the strength of relationships between participants’ residual vision in their blind field and in vivo neurotransmitter concentrations, as well as visually evoked functional magnetic resonance imaging activity in their hMT+. Levels of GABA and glutamate were also measured in a sensorimotor region, which served as a control.

**RESULTS:**

Magnetic resonance spectroscopy-derived GABA and glutamate concentrations in hMT+ (but not sensorimotor cortex) strongly predicted blind-field visual detection abilities. Performance was inversely related to levels of both inhibitory and excitatory neurotransmitters in hMT+ but, surprisingly, did not correlate with visually evoked blood oxygenation level–dependent signal change in this motion-sensitive region.

**CONCLUSIONS:**

Levels of GABA and glutamate in hMT+ appear to provide superior information about motion detection capabilities inside perimetrically defined blind fields compared to blood oxygenation level–dependent signal changes—in essence, serving as biomarkers for the quality of residual visual processing in the blind-field. Whether they also reflect a potential for successful rehabilitation of visual function remains to be determined.

Damage to the primary visual cortex (V1) due to a stroke, results in blindness in the contralateral visual hemifield. Strokes affecting the visual system occur in ≈20% to 57% of stroke survivors and can significantly affect daily living and quality of life.^1^ Despite the high prevalence, there is currently no widely used treatment, and when therapy is recommended it tends to focus on compensating for the visual field deficit through eye movements.^2^ Nonetheless, it is well established that many patients with V1 damage can show significant residual visual processing (also known as blindsight) within their blind fields, particularly for high-luminance, moving stimuli.^3^ Although V1 is damaged, motion stimuli continue to specifically activate the human middle temporal complex (hMT+),^4,5^ an extrastriate cortical region specialized for visual motion processing.^6,7^ This suggests that alternative visual pathways, bypassing V1 and providing direct input to key extrastriate visual areas such as hMT+, may remain intact and might underlie residual vision in the blind field.^8^ Understanding how hMT+ mediates residual processing would be beneficial to selection of future rehabilitation targets.

Over recent years it has become possible to measure the neurochemistry of specific brain regions in vivo in addition to structure and function. Indeed, with strokes that affect the motor system, a reduction in the inhibitory neurotransmitter γ-aminobutyric acid+macromolecules (GABA+) has been related to increased plasticity and rehabilitation success.^9,10^ Although this type of relationship has not been explored in stroke survivors with visual loss, the presence of blood oxygenation level–dependent (BOLD) activity in hMT+ has been related to blindsight performance,^5^ and baseline BOLD signal change in both V1^11^ and hMT+ appears to be predictive of visual rehabilitation success.^12^

In the current study, we asked if the levels of activity and concentrations of key neurochemicals in hMT+ may underlie residual vision in the blind field of those with vision loss from V1 damage. We assessed how GABA+, glutamate (Glx), or BOLD signal changes in the ipsilesional hMT+ relate to individual differences in residual motion detection in the blind field of 19 chronic, unilateral, occipital stroke survivors. In additional to behavioral performance data, we collected structural magnetic resonance imaging (MRI), functional MRI (fMRI), and proton magnetic resonance spectroscopy (MRS) to determine how major inhibitory (GABA+) and excitatory (Glx) neurotransmitter levels, and neural activity in hMT+ related to residual vision. MRS data were collected in hMT+ of the damaged hemisphere and in sensorimotor cortex (M1) of the same hemisphere to use as a control region.

We hypothesized that in stroke survivors with V1 damage (1) lower GABA+, (2) higher Glx, and (3) increased BOLD signal change in hMT+ might underlie superior residual motion processing in the blind field.

## METHODS

### Data Availability

Anonymous MRI and behavioral data will be made available on request.

### Participants

**Figure 1.**
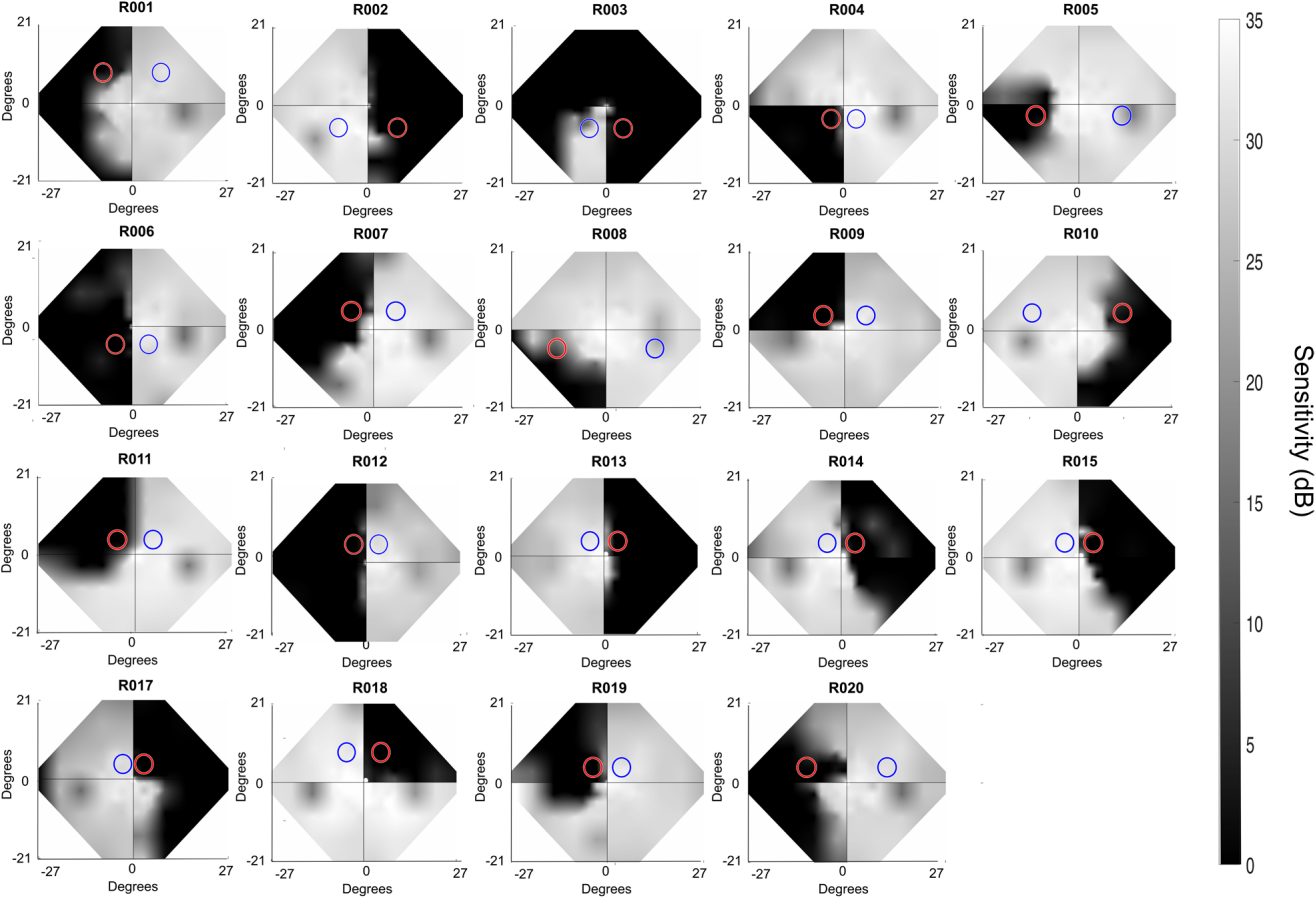
**Composite, interpolated Humphrey visual field plots for all participants.** Grayscale Humphrey-derived visual field sensitivity (24-2 Swedish Interactive Threshold Algorithm [SITA] FAST and 10-2 SITA Standard programs) averaged across the 2 eyes followed by a 2-dimensional interpolation of detection sensitivity in dB (grayscale on right, methods in prior publication^13^). Blind (red circle) and sighted (blue circle) visual field locations for the behavioral and functional magnetic resonance imaging stimulus presentation in each participant are indicated. R011 only completed 24-2 SITA FAST.

Nineteen, otherwise-healthy, stroke survivors with damage to V1 (see Figure S1 for average lesion location) resulting in contralesional visual field deficits (see Figure 1 for participant Humphrey Visual Fields) were recruited to take part in this study (6 female, age 24–74 years, 47.84±15.17; mean±SD; see Table S1 for participant demographics). All participants had sustained damage in adulthood, at least 6 months before the study (44.79±63.50 months; mean±SD). There was no history of diagnosed cognitive or psychiatric disorders (including executive deficits), motor impairments (including those caused by the stroke), previous eye disease, or impairment other than visual loss after stroke (including all forms of visuospatial neglect; see the Supplemental Material for recruitment details).

Participants were asked to refrain from drinking caffeine on the day of the scan and avoid alcohol in the preceding 48 hours. All participants provided informed consent and experiments were conducted in accordance with the ethical guidelines of the Declaration of Helsinki. The study was approved by the University of Oxford Medical Sciences Interdivisional Research Ethics Committee Committee (R60132/RE001).

### Psychophysical Testing

**Figure 2.**
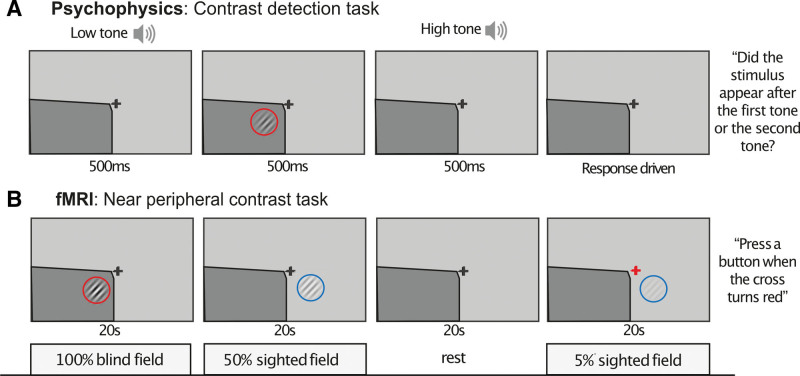
**Schematic of behavioral and functional magnetic resonance imaging (fMRI) tasks. A**, Behavioral contrast detection task: a drifting achromatic obliquely oriented Gabor patch was presented in the Humphrey-defined blind field (dark gray shading). Participants were asked to indicate via keyboard press whether it appeared after the first or second tone. The experiment was also repeated at an equivalent location of the sighted hemifield. **B**, Contrast stimuli and central attentional task performed during fMRI: an achromatic Gabor patch was presented at the same intact and blind-field locations as during behavioral psychophysics in a block design that alternated contrast levels and stimulus presentation with rest periods. To control attention participants pressed a button whenever the central, fixation cross turned from black to red.

A psychophysical task (contrast detection task) was used to measure residual vision in the perimetrically defined blind and sighted fields. This task has been described in detail in prior publications,^12,14^ but involved indicating the time interval in which a drifting, achromatic, Gabor stimulus (diameter=5°; σ=0.8°; stimulus duration=500ms; spatial frequency=1c/°; temporal frequency=10 Hz; viewing distance=42 cm; uniform gray background with a luminance of 16.5 cd/m^2^) of varying contrasts (1%, 5%, 10%, 50%, and 100%) appeared (Figure 2). Since most stroke survivors only show above-chance contrast detection ability in their blind field for high-contrast stimuli,^12,14^ the present experiments only report percent correct performance for high-contrast stimuli (ie, averaged across 50% and 100% contrast stimulus trials). Participants were instructed to fixate on the central cross. Eye position was monitored using an Eyelink 1000 eye tracker (SR Research Limited, Ontario, Canada).

### Imaging Acquisition

#### fMRI Acquisition

MRI scans were acquired on a 3 Tesla MRI scanner Siemens Prisma (Siemens Healthineers AG, Erlangen, Germany) using a 64-channel head/neck coil. A multiband gradient echo sequence^15,16^ was used to acquire fMRI data for the motion localizer and contrast fMRI tasks (see the Supplemental Material for scan details).

Two fMRI tasks were used to (1) localize hMT+ (functional localizer task; see the Supplemental Material for task details), and (2) determine BOLD signal change in hMT+ (contrast fMRI task). Stimuli were generated using MATLAB (2015b or 2018b) and Psychtoolbox (version 3.0.11) and presented on a screen at the back of the MRI scanner bore (viewing distance=127.5 cm). The contrast fMRI task has been described previously,^5,12^ and involved passively viewing a drifting achromatic Gabor patch (same stimulus parameters as psychophysical task) presented at the same location in the blind field and the equivalent location in the sighted field in a block design. In each 20 s block, a single contrast (1%, 5%, 10%, 50%, 100%) or a rest block was presented 8× (2s duration, ISI of 500ms). Each participant completed 3 runs. Participants fixated on a static cross and had to press a button when the cross changed from black to red. Color changes were random and lasted for 300 ms. In 4 cases, the visual field loss was in the far periphery. The fixation cross was then positioned eccentrically to enable the Gabor stimulus to be presented in the blind region.

#### MRS Acquisition

MRS data were acquired in 2 separate scans from 25×25×20 mm^3^ voxels placed over hMT+ and sensorimotor cortex in the damaged (ipsilesional) hemisphere (see the Supplemental Material for positioning details). In the case of bilateral damage, the voxel was located in the hemisphere with the worse visual function (left hemisphere).

GABA+ and Glx were measured from spectra acquired using a locally developed MEGA-PRESS (Mescher-Garwood point resolved spectroscopy) sequence, derived from the Center for Magnetic Resonance Research spectroscopy package (see the Supplemental Material).

In 12 participants, the lesion-affected zone (defined as damaged tissue and fluid-filled space resulting from the stroke) overlapped with the hMT+ voxel, occupying on average 5.68%±9.86% (mean±SD) of the voxel volume. In 7 out of 12 the overlap was >2.5%, with the largest overlap at 36.8% (Figure S1 shows average lesion location). During MRS acquisition participants watched a nature documentary to reduce boredom and sleepiness. Participants were instructed to keep their eyes open throughout, and this was monitored using the eye tracker.

MRS data were excluded from further analyses if (1) Gannet failed to fit the data, (2) the neurochemical concentration value exceeded 3SD from the mean group, and (3) the voxel was incorrectly positioned. All data were included in the hMT+ MRS analysis (N=19). One participant was excluded for each of these reasons for the M1 MRS analysis.

### Imaging Analysis

#### Functional MRI

Preprocessing and statistical analysis of the contrast task fMRI were carried out using Oxford Center for Functional MRI of the Brain software library ([FSL]; version 6.0, http://www.fmrib.ox.ac.uk/fsl; see the Supplemental Material).

To ensure blind-field data were reliable, only participants with BOLD activity in the intact hemisphere for sighted-field stimulation were included. Data in one participant was excluded from fMRI analysis on this basis. Data from 2 participants were not included due to an inability to present stimuli in their blind field, given the eccentricity of the vision loss. Thus 16 participants were included in the fMRI analyses.

Ipsilesional hMT+ masks were created for each participant using the intersection of the motion localizer activity, Jülich histological atlas hMT+ mask, and MRS voxel volume and thresholded to produce a mask guided by the volume of hMT+ in the human literature^12,15^ (see the Supplemental Material for details). The FSL Featquery tool was used to calculate BOLD percentage signal change within the mask.

#### MRS Analysis

MRS data from both voxels were analyzed using Gannet 3.1, an open-source toolbox for analysis of edited MRS data (http://www.gabamrs.com/; see the Supplemental Material). Metabolite concentrations are given as a ratio to total creatine (referred to as GABA+: total creatine [tCr] and Glx:tCr).

Voxels were reconstructed using FSL-MRS^16^ and the proportion of gray matter and cerebral spinal fluid within each voxel was calculated.

### Statistical Analysis

Multiple linear regression and correlation analyses were carried out in R studio (version 4.1.2). Partial correlation analyses were carried out using the *ppcor* package. Multiple linear regression analyses, accounting for age, time since lesion and gray matter volume, and Bonferroni-Holm adjusted *P* values were calculated using the *stats* package. Bayesian statistics were carried out using the *BayesFactor* package. Bayes factors (BF) were interpreted using standard convention.^17^

## RESULTS

Residual blind-field visual performance was measured in each participant using a 2-alternative, forced-choice, psychophysical, contrast detection task (Figure 2), presented within their perimetry-defined blind field. The ability to detect a high-contrast (50% and 100%), moving, achromatic Gabor patch varied across participants (mean±SD accuracy=77±19.6% correct; range=30%–100% correct). Twelve participants performed significantly above chance, determined using a statistical threshold of *P*<0.05 and a cumulative binomial distribution.^14^ Percentage correct on this contrast detection task was used as the measure of residual vision for all subsequent analyses.

**Figure 3.**
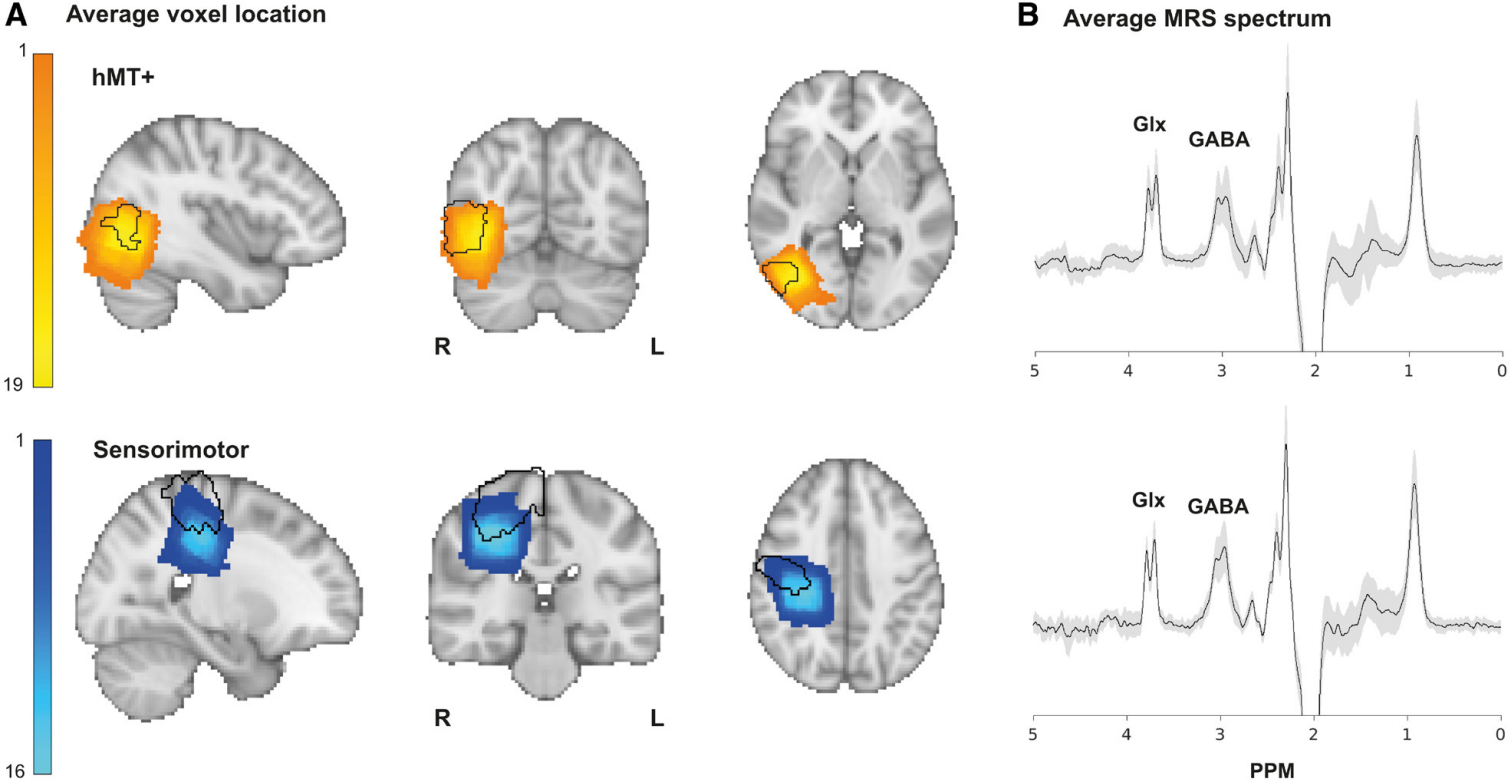
**Voxel placement and spectra for human middle temporal complex (hMT+) and sensorimotor cortex (M1) voxels in the ipsilesional hemisphere. A**, Average placement for the visual motion area (hMT+; n=19) and sensorimotor cortex (M1; n=16) proton magnetic resonance spectroscopy (MRS) voxels. The MRS voxels of participants who had left hemisphere strokes were flipped so that all voxels were shown in the same hemisphere across participants. Yellow (hMT+, **top**) and blue (M1, **bottom**) heat maps represent the group voxel placement. Black outlines represent Jülich histological atlas masks for hMT+ visual area (hMT+/V5) and M1. **B**, Average spectra for the hMT+ (**top**; n=19) and M1 voxels (**bottom**; n=16). Black lines represent the average spectrum across all participants, with gray representing the SD of the spectra. GABA indicates γ-aminobutyric acid; Glx, glutamate+glutamine; GABA+, GABA+macromolecules; L, left; PPM, parts per million; and R, right.

MRS was used to measure GABA+:tCr and Glx:tCr in 2 voxels: one in hMT+ and a control voxel in M1. Figure 3A shows the average location of the voxels across participants. The voxel was always placed in the ipsilesional hemisphere but is illustrated in the right hemisphere. The mean MRS spectrum for each voxel across participants is shown in Figure 3B, with SD indicated by the gray region. GABA+:tCr concentration ratios averaged 0.10±0.02 in hMT+ and 0.14±0.04 in M1. Glx:tCr was 0.07±0.02 in hMT+ and 0.08±0.02 in M1. These measures were compared with percentage correct on the moving contrast detection task.

**Figure 4.**
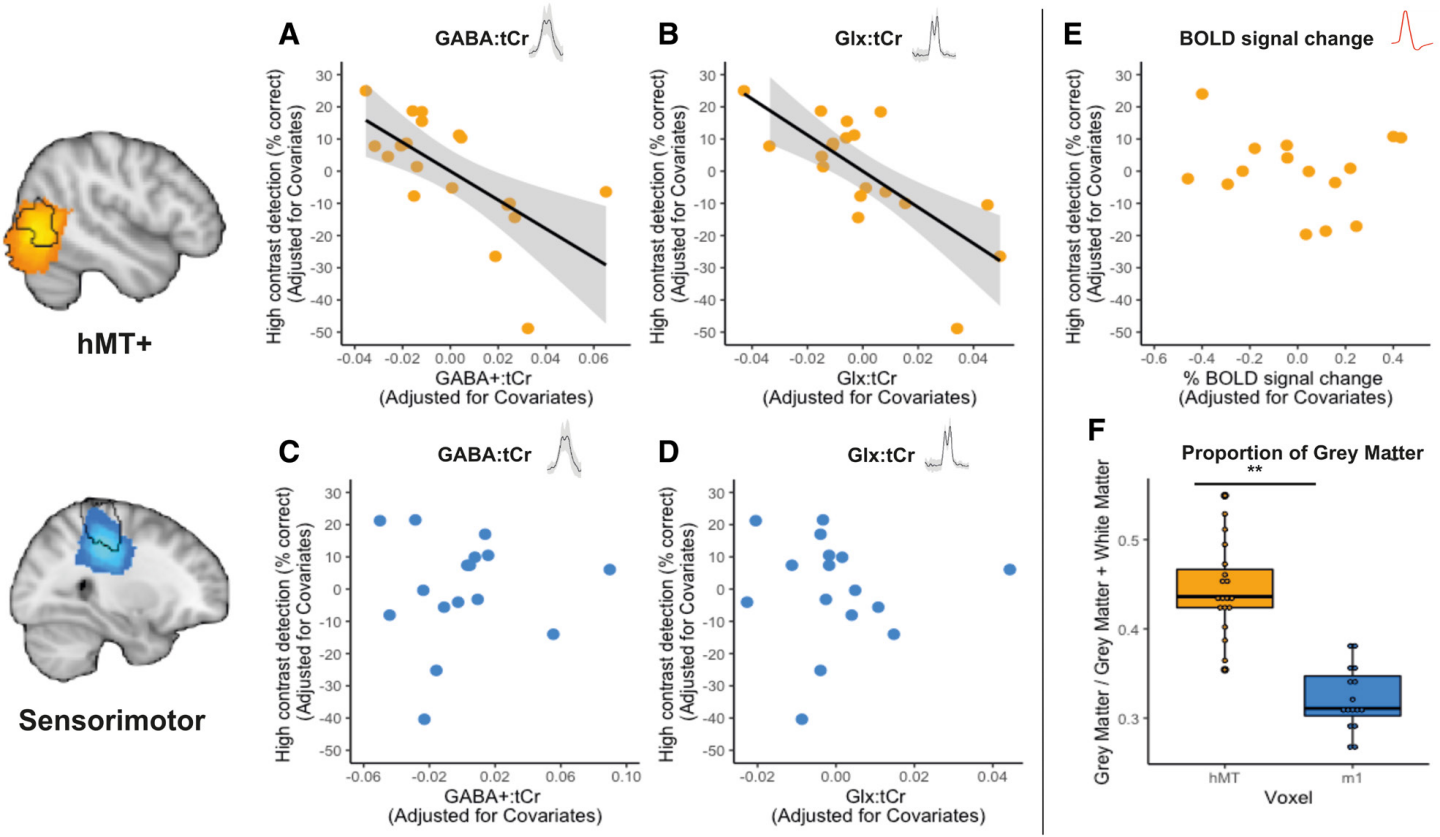
**Plots showing the relationship between contrast detection in the blind field, neurochemicals, and blood oxygenation level–dependent (BOLD) signal change. A** through **E**, Partial regression plots showing the relationship between contrast detection in the blind field and (**A**) γ-aminobutyric acid+macromolecules (GABA+): total creatine (tCr) in ipsilesional human middle temporal complex (hMT+), (**B**) glutamate+glutamine (Glx):tCr in ipsilesional hMT+, (**C**) GABA+:tCr in ipsilesional sensorimotor cortex (M1), (**D**) Glx:tCr in ipsilesional M1, (**E**) percentage BOLD signal change in ipsilesional hMT+. Lower concentration of GABA+:tCr and Glx:tCr was related to better performance on contrast detection within the blind field. There was also no significant relationship between residual vision in the blind field and concentrations of GABA+ or Glx in M1. There was no relationship between percentage BOLD signal change in hMT+ and contrast detection within the blind field. Significant regression lines are plotted in black, with standard errors in gray. **F**, Box plot indicating proportion of gray matter in each voxel. The hMT+ voxel contained significantly more gray matter than the M1 voxel.

There was substantial evidence for a negative relationship between GABA+:tCr measured within the hMT+ voxel and behavioral contrast detection performance using both conventional (Figure 4A: adjusted R^2^=0.41, F[4,14]=4.08; Holm-Bonferroni corrected *P*=0.011, β=−0.61) and Bayesian statistics (BF_10_=3.53). Comparable, strong evidence for a negative relationship was also present between Glx:tCr and behavioral performance (Figure 4B: adjusted R^2^=0.53, F[4,14]=6.13; Holm-Bonferroni corrected *P*=0.0009, β=−0.68; BF_10_=12.42). There was no strong evidence for a relationship between contrast detection performance and either GABA+ (Figure 4C: adjusted R^2^ value=0.12; F(4,11)=1.51; *P*=0.896; β=0.04; BF_10_=0.50) or Glx concentrations in the M1 voxel (Figure 4D: adjusted R^2^ value=0.12; F[4,11]=0.35; *P*=0.897; β=−0.04; BF_10_=0.50).

We also found a significant, positive correlation between GABA+ and Glx concentrations in hMT+ (correlation coefficient=0.78, *P*=0.0001, Spearman correlation), but not in the M1 voxel (correlation coefficient=0.20, *P*=0.471, Spearman correlation). Patient age, months since lesion, and proportion of gray matter volume within the voxel were controlled in all analyses. When a ratio of Glx/GABA+ was calculated to estimate excitatory-inhibitory balance, there was no correlation with behavior (R=−0.33; *P*=0.172).

Percentage BOLD signal change in hMT+, measured while participants viewed a moving, achromatic, Gabor patch of high contrast in their perimetry-defined blind fields, was compared with rest periods (Figure 2). There was no strong evidence for a relationship between visually induced BOLD signal changes during fMRI and contrast detection performance (Figure 4E: adjusted R^2^=0.41; F[3,11]=4.24; Holm-Bonferroni corrected *P*=0.577, β=−0.13; BF_10_=0.47).

## DISCUSSION

The present study shows, for the first time, that MRS-derived measures of neurochemistry in hMT+ are directly related to individual differences in the ability to detect drifting, high-contrast, Gabor patches inside perimetrically defined blind fields in patients with chronic stroke with V1 damage. Specifically, participants with lower levels of both GABA+ and Glx in hMT+ were better at performing this contrast detection task in their blind field than those with higher levels of both neurotransmitters. In contrast, there was no relationship between BOLD signal change in hMT+ when exposed to the same stimulus, and blind-field ability to detect it during psychophysical testing.

### Lower Levels of GABA+ and Glx Link to Greater Residual Vision in the Blind Field After Occipital Stroke

In chronic motor stroke survivors, GABA+ and Glx decrease in M1 relative to controls, and greater decreases in GABA+ correlate with improvement due to rehabilitation.^9^ Here, we posited that, as in the motor system,^18^ decreased inhibitory activity in the residual visual system long after occipital stroke—especially in intact extrastriate visual areas—could play a significant role in supporting neuroplastic mechanisms. In the healthy visual system, Frangou et al^19^ found that reduced GABA in the occipital lobe was related to the ability to detect targets in clutter, while GABA was increased when discriminating fine feature differences. The psychophysical task used to measure residual perceptual abilities in the blind field required detection of a moving Gabor patch rather than fine feature discrimination. Given that the V1-damaged visual system appears to suffer from increased levels of internal processing noise,^20^ a plausible hypothesis emerging from the present results is that GABAergic disinhibition in the motion processing visual cortical area hMT+ may work to enhance target detection by increasing signal-to-noise. The current findings are also consistent with Lunghi et al^21^ who showed that decreased GABA following short-term monocular deprivation correlated with greater plasticity of binocular vision in the healthy system.

Of note, we were surprised to find that reduced Glx levels in hMT+ were related to superior motion detection in the blind field, despite initially hypothesizing the opposite effect. Research from the animal literature indicates that excitatory and inhibitory activity must remain in balance in the brain, with individual neurons receiving roughly equal levels of excitation and inhibition.^22^ This mechanism is necessary to prevent the system from becoming overactive (high excitation; low inhibition) or overly suppressed (low excitation; high inhibition). Previous MRS studies in humans indicate that the balance between excitation and inhibition depends on the anatomic region being measured. Studies in humans have found a positive relationship between GABA+ and Glx in the medial parietal cortex,^23^ but not early visual cortex.^24^ However, a subsequent study by the same authors using a large dataset found a positive relationship between GABA+ and Glx in the early visual cortex but not in the prefrontal cortex.^25^ Moreover, evidence from the same anatomic location as the current study, extrastriate hMT+,^26^ indicates a positive relationship between GABA+ and Glx. We originally hypothesized that increased plasticity after stroke, and superior residual vision, would be associated with lower GABA+. If this were the case, an equivalent reduction in Glx would be required to maintain the inhibitory-excitatory balance. In the current study, GABA+ and Glx were highly correlated in hMT+, and when the variance between GABA+ and behavior was controlled, the relationship with Glx disappeared. However, our control analyses indicated that the relationship was unlikely to be driven by data quality (specifically linewidth). These results further suggest that the 2 neurochemicals are not functionally independent and that changes in both neurochemicals affect behavior. Although it is not possible to determine whether the differences observed are driven by GABA+ or Glx, we theorize that the higher levels of Glx and GABA+ in those who have the least residual vision may reflect reduced plastic potential of hMT+, driven by higher GABA+. However, future studies are needed to explore how these neurochemicals relate to training-induced recovery.

### No Relationship Between BOLD Response and Residual Vision

Although prior studies investigating residual vision or blindsight reported activity in hMT+ when moving stimuli were passively presented in the blind field,^4,5,27–29^ they did not relate the extent of activity to performance of a detection task with respect to these stimuli, as done here. Nonetheless, they provided evidence that the pathway between the dorsal lateral geniculate nucleus and hMT+ likely carried relevant visual information.^14,30,31^ This motivated our hypothesis that V1-independent residual vision for moving Gabor patches might be linked to increased BOLD signal in hMT+. However, no such relationship was found in the present study.

BOLD signal changes are thought to indirectly reflect changes in neural activity through neurovascular coupling; however, the relationship between neural activity and the hemodynamic response depends on a combination of cerebral blood flow, blood oxygenation levels, and blood volume, some of which may be abnormal in stroke survivors. In strokes affecting the motor system, the BOLD signal is delayed and of lower amplitude in the affected and unaffected sensorimotor cortex.^32^ Furthermore, multimodal imaging studies in motor stroke survivors have shown the presence of neuronal responses measured with magnetoencephalography^33^ and transcranial magnetic stimulation^34^ even in the absence of a BOLD response. The lack of relationship between motion detection and BOLD signal evoked by passive exposure of a motion stimulus while performing an attentionally demanding task at fixation in the current study may, therefore, not reflect a lack of neuronal activity in hMT+ in stroke survivors with V1 damage.

Although there was no relationship between performance and BOLD activity in this and previous studies, recent work found that baseline BOLD activity in hMT+^12^ and perilesional V1,^11^ and BOLD resting-state connectivity between the anterior precuneus and the occipital pole network^35^ correlate with the extent of improvement following rehabilitation. This suggests that the relationship between the BOLD signal and residual vision is likely to be complex.

### Limitations

The extensive time required to setup and acquire MRS data from each voxel (≈20 minutes) meant that we were unable to collect neurotransmitter concentration data from >2 voxels. Although it would have been beneficial to also collect data from hMT+ in the intact hemisphere, there is a known interhemispheric interaction between hMT+ after damage to V1^36^ meaning it would not be a pure control structure.

The data were collected during the baseline session of a rehabilitation study, so there is no control group with normal visual function. Thus, while we can correlate neurochemical concentrations with residual visual detection in the blind field, we cannot determine whether there is a change in concentration as a result of the occipital stroke itself. This is compounded by the lack of any normative data for neurochemical concentrations in hMT+ and the variability in concentrations across different brain regions, individuals, or time.

A major challenge in all stroke-related research is the heterogeneity among stroke survivors. Damage caused by stroke is highly variable, even when it is confined to a single area, such as the occipital cortex, and is dependent on factors such as age and type of stroke.^37^ Much of the research investigating residual vision after damage to V1 has focused on individual case studies.^38,39^ When larger sample sizes are used, patients with a variety of causes, including stroke, trauma, or tumor resections,^5,14^ are often included. In the current study, as in several more recent publications,^35,40^ we aimed to minimize the variability by limiting our inclusion criteria to stroke damage in adulthood. However, to achieve the sample size for the study, it was not possible to control for the type of stroke (7 hemorrhagic; 10 ischemic; 2 unknown). The large range of age (24–74 years) and time since stroke (6–297 months) is a further challenge. Although controlled for in all analyses, the limited sample size means it is not possible to fully account for any effects on neurochemistry. It is, therefore, possible that the cause of stroke, age, or time since lesion may also impact on residual vision and neurochemistry, and future studies with an increased (or homogeneous) sample will be needed to investigate this further.

Although MRS is a valuable technique that allows us to quantify measurements of neurochemicals in vivo, there are several limitations when interpreting the relationship between neurochemicals and behavior. First, MRS measures total concentration of each neurochemical within a defined area of cortex (≈8 cm^3^) averaged across the duration of the scan (≈8 minutes). At these spatial and temporal scales, it is not possible to obtain a direct measure of synaptic activity, nor to determine where within the voxel the neurochemical concentration may vary. Moreover, using GABA-editing MEGA-PRESS sequences in a 3 Tesla MRI scanner it is not possible to separate the resonances of glutamate and glutamine (resulting in the combined measure of Glx) and the GABA signal is contaminated by macromolecules^41^ and homocarnosine.^42^ Thus, MRS-derived quantifications of GABA and glutamate may be driven by these alternative signals. This could account, at least in part, for the lack of relationship between the ratio of Glx/GABA+ and behavior in the current study. It is possible that acquiring at higher field strengths to separate glutamate and glutamine, or using macromolecular suppression to reduce contamination of GABA, could produce different results. At ultra-high field strength (7 Tesla MRI scanner), where glutamate and glutamine can be separated, a recent study found evidence for a relationship between GABA+ and glutamate in both prefrontal and occipital cortex.^25^ However, they only found a relationship with Glx in the occipital lobe and not the prefrontal cortex, pointing towards regional differences between measures of glutamate and Glx.

MRS-derived measures of GABA and glutamate are usually quantified as a ratio relative to another neurochemical (eg, creatine) or endogenous reference such as water. The specific reference used varies, with creatine, N-acetylaspartate, or an unsuppressed water signal commonly used. Here we referenced GABA and glutamate to creatine, a commonly used method in MRS studies.^9,43,44^ Creatine is an organic compound found in high concentration in the brain, believed to have a role in energy storage, high-energy phosphate shuttle, osmoregulation, and neurotransmission.^45^ Although creatine is not a reliable reference for MRS in acute stroke,^46^ it has been used for most MRS studies in patients with chronic stroke^9,43,44^ as it largely removes the need to control for cerebral spinal fluid^47^ and tissue composition, while also controlling for variation due to instrumentation as the reference metabolite experienced the same acquisition environment. Importantly, in the current study, the GABA+ and Glx signals referenced to water were significantly correlated with the extent of overlap between the stroke-induced fluid-filled zone and the MRS voxel. This was not the case when they were referenced to creatine, suggesting that water did not provide a suitable reference. This analysis does not guard against the possibility of creatine changing differently to GABA/Glx in lesioned tissue.

### Conclusions

In conclusion, this is the first study to explore neurochemistry in the visual system of chronic stroke survivors with visual loss. Lower levels of GABA+ and Glx in the motion-selective extrastriate region hMT+ are linked to better residual visual detection performance in the blind field. Despite the lack of normative data, the presence of this correlation suggests that the neurochemistry of hMT+ is altered to different degrees in different patients with V1 damage. Importantly, neurochemistry in hMT+ may provide useful information about neural functioning not captured by BOLD signal change. It may thus serve as a biomarker for enhanced visual processing abilities inside the blind field which, in turn, may reflect increased capacity for neurorehabilitation.

## ARTICLE INFORMATION

### Acknowledgments

The authors thank our participants for generously giving up their time to participate. Additionally, the radiographers David Parker, Michael Sanders, Nicola Aikin, and Juliet Semple, optometrist Patsy Terry, and optometric technician Charlene Hennesey for assistance with training and data collection. Also, Ione Fine for her comments on the article.

### Sources of Funding

The project was funded by European Research Council grant to Dr Tamietto (prot. 772953) and a British Medical Association Foundation John Moulson Grant to H. Bridge. H.E. Willis was supported by the Medical Research Council and a Waverley Scholarship from The Queen’s College, Oxford. I.B. Ip was supported by a Royal Society Dorothy Hodgkin Research Fellowship (DHF\R1\20114112). The Wellcome Center for Integrative Neuroimaging is supported by core funding from the Wellcome Trust (203139/Z/16/Z). For the purpose of Open Access, the author has applied a CC BY public copyright license to any author-accepted article version arising from this submission.

### Disclosures

None.

### Supplemental Material

Supplemental Methods

STROBE Checklist

Minimum Reporting Standards for MRS Checklist

## Supplementary Material
