# Supplementary material for: GABA and Glutamate in hMT+ Link to Individual Differences in Residual Visual Function After Occipital Stroke
Source: Stroke. 2023 Jul 21;54(9):2286–95. doi: 10.1161/STROKEAHA.123.043269 (PMC10453332; doi:10.1161/STROKEAHA.123.043269)
Supplement: Supplementary file 1 [file str-54-2286-s001.pdf]

SUPPLEMENTARY MATERIAL

Expanded materials and methods

Participants

Most participants were recruited (i) after directly contacting study authors following internet searching, (ii) via word of mouth in ophthalmology departments or (iii) had previously participated in studies. All participants who met the inclusion criteria and were within reasonable travelling distance were included in the study. Participants were not required to have a previous brain scan and therefore no exclusion based on lesion size was made.

Table S1. Participant demographics

Participant	Sex	Age	Stroke Cause	Time since stroke (months)	Visual field defect side
R001	m	54	Ischemic stroke	68	Left
R002	f	28	Ischemic stroke	39	Right
R003	m	33	Hemorrhagic stroke	58	Both
R004	f	51	Ischemic stroke	31	Left
R005	m	61	Hemorrhagic stroke	21	Left
R006	m	24	Hemorrhagic stroke	13	Left
R007	m	47	Hemorrhagic stroke	7	Left
R008	m	45	Ischemic stroke	32	Left
R009	m	35	Hemorrhagic stroke	25	Left
R010	m	63	Ischemic stroke	47	Right
R011	m	64	Ischemic stroke	7	Left
R012	m	74	Ischemic stroke	58	Left
R013	m	71	Unknown	26	Right
R014	f	34	Hemorrhagic stroke	30	Right
R015	f	37	Hemorrhagic stroke	29	Right
R017	m	66	Ischemic stroke	6	Right
R018	m	35	Ischemic stroke	26	Right
R019	f	41	Ischemic stroke	31	Right
R020	f	46	Unknown	297	Right

Functional motion localiser task

A functional motion localiser task was used to identify area hMT+ during the scan in each participant to guide the ^{1}H-MRS voxel placement. White dots were presented within two rectangles either side of fixation (width 10°, number of dots = 400, dot size =0.12°, dot speed=15°/s) on a uniform grey background, with a central red fixation cross. The block design (15s) presented 4 alternations of stationary and coherently moving blocks (15°/s per block, total run duration=120s). The direction of movement (45, 90, 135, 180, 225, 270°) varied randomly every ~2.5 seconds.

fMRI acquisition

Multiband gradient echo sequence was used to acquire fMRI data for the motion localiser (2.4 mm isotropic resolution, TR=1000ms, TE=39.0ms, flip angle=52°, multi-band factor=6) and contrast fMRI task (2mm isotropic resolution, TR=1050 ms, TE=37.0 ms, flip angle=60°, multi-band factor=6). A high-resolution whole-head T1-weighted isotropic MPRAGE anatomical image (1x1x1mm^3, TR=1900ms, TE=3.97ms, field-of-view = 192 mm x 192 mm, flip angle = 8°) was also collected for each participant.

^{1}H-MRS acquisition

MEGA-PRESS sequence (https://www.cmrr.umn.edu/spectro/) with the following parameters: 320 transients (160 per editing condition); 2048 data points; 4000 Hz spectral width; TR=1500 ms; TE=68 ms; VAPOR and dual-band editing pulse water suppression; 22.3 ms editing pulse with a 53 Hz bandwidth, centered at 1.9 ppm ("on" condition) and at 7.5 ppm ("off" condition) in alternating transients; 16-step phase cycling. For each voxel, a single transient was also collected with water suppression disabled (TR=2500 ms). GRE shimming was used to ensure that vendor-reported full-width-at-half-maximum (FWHM) were below 20Hz, and that water-unsuppressed MRS-measured FWHM were <12Hz. Figure 2 shows the average voxel position and spectra across participants.

MRS data were acquired in two voxels of interest. The main voxel of interest was placed using the T1-weighted structural image in extrastriate visual motion area hMT+. It was positioned parallel to the lateral cortical surface[17] and then adjusted based on

the activity from the functional motion localiser. The control sensorimotor voxel (M1) was centered on the “hand knob” in the ipsilesional central sulcus and positioned parallel to the dorsolateral cortical surface[18]. Data acquisition from each voxel required around 20 minutes of scanning including setup. Thus, it was not possible to also acquire data from the contralesional hemisphere or from additional brain areas, given patient tolerance and timing constraints.

fMRI analysis

fMRI analysis was carried out using FSL. The following steps were used: 1) brain extraction with BET, 2) motion correction with MCFLIRT, 3) distortion correction using a field map 4) spatial smoothing using 5mm Gaussian kernel and 5) high pass temporal filtering (cut off 90%). Functional images were registered to structural images using FLIRT (BBR method). To allow for easier comparison, structural and functional images from all left hemisphere stokes were flipped (fslswapdim tool in FSL) to simulate right hemisphere strokes[14]. Using FEAT the visual contrasts (1%, 5%, 10%, 50% and 100%) were included as explanatory variables. However, as in the behavioral data, the contrast of interest was set to high contrast (50% and 100%) compared with rest for sighted and blind field presentation within the GLM model.

Individualized hMT+ masks were created for each participant using the intersection of the motion localiser activity, Jülich histological atlas hMT+ mask and ^{1}H-MRS voxel volume. The Jülich histological atlas implemented in FSL contains probabilistic definitions of brain regions that were identified based on post-mortem myelo- and cyto-architecture in 10 brains and transformed to standard space. These masks were thresholded to produce a mask between 1500 and 2000 mm^3 guided by the volume of hMT+ in the human literature[14,17].

^{1}H-MRS analysis

^{1}H-MRS data were analyzed with Gannet 3.1. Pre-processing included frequency and phase correction in the time domain, 3Hz exponential line broadening and zero-filled by a factor of 16. A five-parameter Gaussian model was used to fit the GABA peak between 2.19 and 3.55ppm. GABA and Glx peaks were quantified relative to total

creatine (tCr). Creatine was calculated from the total creatine (tCr) integral of a two-Lorentzian model of creatine and choline metabolites in the OFF spectrum.

Average Lesion Location

The binarized lesion mask for each participant was transformed into standard space and then lesions from all participants were summed to produce an image with the magnitude indicating the number of participants with overlapping lesions. The rainbow heatmap in Supplementary Figure 1 shows the lesion overlap with definitions of hMT+ and V1 outlined.

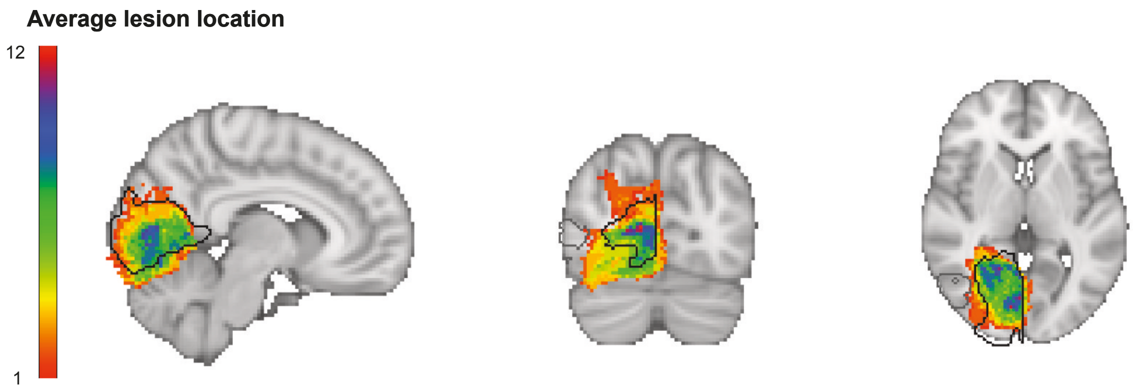

Figure S1. Average lesion location (N=19; rainbow heatmap) standardized across patients to have the lesion in the right hemisphere displayed on the MNI-152 standard brain. These heatmaps indicate that the main overlap of lesions across participants was centered on the calcarine sulcus, the anatomical location of V1. Jülich regions of interest of hMT+ (grey) and V1 (black) are indicated.

Data quality

The output of Gannet was visually inspected for evidence of lipid contamination or poor water suppression. Fit error (under 15%) and full-width at half-maximum (FWHM) were calculated and all values in both voxels were comparable to previous reported values (see Supplementary Table 2).

Table S2: Data quality checks

Voxel	*Mean GABA fit error (sd)*	*Mean Glx fit error (sd)*	*Mean GABA FWHM (sd)*	*Mean Glx FWHM (sd)*
hMT+	6.98 (1.75)	6.06 (1.74)	22.37 (2.85)	15.15 (3.89)
M1	7.35 (1.77)	*6.92 (1.18)*	22.65 (2.36)	13.28 (2.00)
t-tests	t=0.62, p=.539	t=1.72, p=.094	t=0.32, p=.748	t=-1.83, p=.078

As GABA+ and Glx were both negatively related to behavior, it is possible that the relationship might be due to shared variance, rather than changing metabolite pools. One potential confounding variable is spectral quality. Linewidth is often used as a measure of signal quality as larger linewidths are generally associated with lower quality spectral acquisition[48.] To ensure that relationships between behavior and GABA+ and Glx were not driven by spectral quality, we explored the relationship between behavior and tNAA linewidth, creatine linewidth and fit error. There was no significant correlation between behavior and tNAA linewidth *(R=-0.34, p=0.153;* BF_{+0}*=0.86),* water linewidth *(R=-0.29, p=0.216;* BF_{+0}*=0.71),* creatine linewidth *(R=-0.22; p=0.355;* BF_{+0}*=0.55),* GABA+ fit error *(R=0.39; p=0.093;* BF_{+0}*=1.16)* or Glx fit error *(R=0.19; p=0.441;* BF_{+0}*=0.50).* Additionally, to further determine whether the associations between each metabolite and behavior was driven by common variance, we modelled the relationship between GABA and behavior using a linear regression and then looked at the correlation between the residuals from this model and Glx. No significant correlation was found between the two *(R=-0.29; p=0.225;* BF_{+0}*=0.69).* To conclude, these control analyses confirmed the relationship between GABA+ and Glx and behavior is not driven by these confounds. However, the Bayes factors indicate that there is only weak support for the null hypothesis.

We used total creatine as a reference metabolite rather than water for several reasons, with the principal one being the overlap of the lesion-affected zone with the MRS voxel in some participants. To investigate the effects of the fluid in the lesion-affected zone we measured the correlation between the percentage overlap with the voxel and GABA+ and Glx referenced to both tCr and water (w). We found that there was no relationship between GABA+:tCr (r=-0.32; p=0.185) or Glx:tCr (r=-0.23; p =0.344) and percentage overlap. However, when the two metabolites were referenced to water, the amount of overlap was significantly correlated with the ratios in both cases (GABA+:w – r=-0.63; p =0.004; Glx:w – r =-0.57; p =0.010). Therefore, we did not use water as a reference signal in this analysis.

Covariates

To ensure that controlling for covariates was not driving the results in these analyses, we also computed simple linear regressions between behavior and GABA+ and Glx. These analyses indicate a significant relationship between behavior and GABA+ (*adjusted* R^2*=0.26; F(1,17)=7.45; p=0.01)* and Glx (*adjusted* R^2*=0.36; F(1,17)=11.17; p=0.004)* in hMT+, even when not controlling for potential covariates.

Pre-registration

Pre-registration of this study can be found at https://osf.io/ry2eg. In the pre-registration of this study, we hypothesized a correlation between BOLD signal in hMT+ and GABA+ and Glx concentrations. However, given the lack of relationship between BOLD signal and behavior, we did not investigate the relationship between BOLD signal and neurochemicals further.

STROBE Statement—checklist of items that should be included in reports of observational studies

	Item No	Recommendation	Line No
**Title and abstract**	1	(*a*) Indicate the study's design with a commonly used term in the title or the abstract	1-2
		(*b*) Provide in the abstract an informative and balanced summary of what was done and what was found	29-58
**Introduction**			
Background/rationale	2	Explain the scientific background and rationale for the investigation being reported	60-95
Objectives	3	State specific objectives, including any prespecified hypotheses	97-99
**Methods**			
Study design	4	Present key elements of study design early in the paper	85-95
Setting	5	Describe the setting, locations, and relevant dates, including periods of recruitment, exposure, follow-up, and data collection	supplementary
Participants	6	(*a*) *Cohort study*—Give the eligibility criteria, and the sources and methods of selection of participants. Describe methods of follow-up<br>*Case-control study*—Give the eligibility criteria, and the sources and methods of case ascertainment and control selection. Give the rationale for the choice of cases and controls<br>*Cross-sectional study*—Give the eligibility criteria, and the sources and methods of selection of participants	104-113
		(*b*) *Cohort study*—For matched studies, give matching criteria and number of exposed and unexposed<br>*Case-control study*—For matched studies, give matching criteria and the number of controls per case	
Variables	7	Clearly define all outcomes, exposures, predictors, potential confounders, and effect modifiers. Give diagnostic criteria, if applicable	131-229
Data sources/ measurement	8*	For each variable of interest, give sources of data and details of methods of assessment (measurement). Describe comparability of assessment methods if there is more than one group	131-229
Bias	9	Describe any efforts to address potential sources of bias	234-235
Study size	10	Explain how the study size was arrived at	104-113
Quantitative variables	11	Explain how quantitative variables were handled in the analyses. If applicable, describe which groupings were chosen and why	
Statistical methods	12	(*a*) Describe all statistical methods, including those used to control for confounding	232-237
		(*b*) Describe any methods used to examine subgroups and interactions	
		(*c*) Explain how missing data were addressed	197-202; 211-214
		(*d*) *Cohort study*—If applicable, explain how loss to follow-up was addressed<br>*Case-control study*—If applicable, explain how matching of cases and controls was addressed<br>*Cross-sectional study*—If applicable, describe analytical methods taking account of sampling strategy	
		(*e*) Describe any sensitivity analyses	

**Results**			
Participants	13*	(a) Report numbers of individuals at each stage of study—eg numbers potentially eligible, examined for eligibility, confirmed eligible, included in the study, completing follow-up, and analysed	104-113; 197-202; 211-214
		(b) Give reasons for non-participation at each stage	197-202; 211-214
		(c) Consider use of a flow diagram	
Descriptive data	14*	(a) Give characteristics of study participants (eg demographic, clinical, social) and information on exposures and potential confounders	104-113
		(b) Indicate number of participants with missing data for each variable of interest	197-202; 211-214
		(c) *Cohort study*—Summarise follow-up time (eg, average and total amount)	
Outcome data	15*	*Cohort study*—Report numbers of outcome events or summary measures over time	*240-313*
		Case-control study—Report numbers in each exposure category, or summary measures of exposure	
		Cross-sectional study—Report numbers of outcome events or summary measures	
Main results	16	(*a*) Give unadjusted estimates and, if applicable, confounder-adjusted estimates and their precision (eg, 95% confidence interval). Make clear which confounders were adjusted for and why they were included	*240-313*
		(*b*) Report category boundaries when continuous variables were categorized	
		(*c*) If relevant, consider translating estimates of relative risk into absolute risk for a meaningful time period	
Other analyses	17	Report other analyses done—eg analyses of subgroups and interactions, and sensitivity analyses	
**Discussion**			
Key results	18	Summarise key results with reference to study objectives	319-326
Limitations	19	Discuss limitations of the study, taking into account sources of potential bias or imprecision. Discuss both direction and magnitude of any potential bias	408-476
Interpretation	20	Give a cautious overall interpretation of results considering objectives, limitations, multiplicity of analyses, results from similar studies, and other relevant evidence	328-404
Generalisability	21	Discuss the generalisability (external validity) of the study results	328-476
**Other information**			
Funding	22	Give the source of funding and the role of the funders for the present study and, if applicable, for the original study on which the present article is based	496-502

*Give information separately for cases and controls in case-control studies and, if applicable, for exposed and unexposed groups in cohort and cross-sectional studies.

Note: An Explanation and Elaboration article discusses each checklist item and gives methodological background and published examples of transparent reporting. The STROBE checklist is best used in conjunction with this article (freely available on the Web sites of PLoS Medicine at http://www.plosmedicine.org/, Annals of Internal Medicine at http://www.annals.org/, and Epidemiology at http://www.epidem.com/). Information on the STROBE Initiative is available at www.strobe-statement.org.
